# The Air of the Bed-Room

**Published:** 1889-03

**Authors:** 


### The Air of the Bed-Room.

The season is now at hand when the problem of ventilation is most important and most difficult of solution. Cold outside, and attempts to maintain a warm temperature inside our houses combine to make living dangerous to all but the most robust constitutions; and it is not easy to arrange matters so that comfort and safety to health shall go hand in hand. This is especially true in regard to the management of the air in bed-rooms.

There are those who still cling to the idea, which no intelligent medical man now holds, that a cold bed-room is a promoter of health and vigor. Recent studies of the conditions which favor the production of diseases of the lungs leave no doubt that sleeping in a cold room is a most dangerous practice. There are those who can stand it; but it is only because they have unusual powers of resistance. The great mass of mankind, old and young, need to have their bodily temperature carefully maintained at night, and the air they then breathe at about the same temperature as that of the air they breathe by day. Indeed, if there is any difference, it should be that the air which surrounds them when they sleep is somewhat warmer than that in which they move about by day.

For many years those who taught hygiene in popular treatises argued from certain assumptions which had an appearance of soundness, but which were actually unsound, and directly contrary to experience. The fear of what was called foul air led them to underrate the dangers of cold air; and in attempting to escape the former they ran right into the latter. It is strange that these well-meaning teachers failed to see the contradiction to their theories which is furnished by the inhabitants of very cold regions, and that they did not ask themselves why men, women, and children, who spent the day in the pure and cold air of the Arctic regions and the night in the hot and close atmosphere of their huts, were exceptionally free from diseases of the lungs.

In our day the lesson this fact teaches is better understood, and we know that draughts and a chilly atmosphere in the so-called temperate regions are more dangerous to life and health than foul air seems to be in the frigid zones.

All this is not intended as a defense of foul air; but rather as a warning against cold air. And, those who give advice as physicians may bear it in mind when consulted in regard to the management of living rooms and sleeping rooms in our cold winters. For bed-rooms especially, they may without fear of error recommend a temperature which shall not put any strain upon immature or frail constitutions, and warn their patients against any system of ventilation which involves a cold bed-room.

Bed-rooms should not be over-heated, but they should never be under-heated. A temperature of about 60° is probably a safe one for sleeping apartments. Ventilation should never be secured by means of open windows, but rather by partly open doors, with precautions to prevent a draught across a bed, or a current of air from a room in which there is any known source of contamination. If a fresher air is desired than many houses have at about the usual hour for retiring, it may be secured by freely ventilating the sleeping-rooms shortly before they are to be occupied, and the rest of the house for a few minutes before the last member of the family goes to bed.

The details of this method can easily be worked out by any intelligent man or woman, and we believe it will furnish a trustworthy means of securing a safe atmosphere as well as a comfortable one.

The matter is so important that we would gladly devote more space to its consideration; but we must leave it here to the consideration of our readers.

---

Death of a Manufacturer of Medical Preparations.—Mr. J. W. Lambert, of St. Louis, the well-known inventor and manufacturer of a number of medical preparations, one of which, and the most noted, was listerine, died January 4. He was but thirty-six years of age, yet he had acquired a most extensive reputation. The company of which he was president will suffer in his decease a great loss.

